# Supplementary material for: Comparative genomics provides new insights into the diversity, physiology, and sexuality of the only industrially exploited tremellomycete: Phaffia rhodozyma
Source: BMC Genomics. 2016 Nov 9;17:901. doi: 10.1186/s12864-016-3244-7 (PMC5103461; doi:10.1186/s12864-016-3244-7)
Supplement: Additional file 6: — List of orphan genes with links to PFAM (related to Additional file 1: Table S1). (ZIP 1428 kb) [file 12864_2016_3244_MOESM6_ESM.zip › BLAST_HTML_FTR/G04373_P.html]

BLAST Search Results


```
BLASTP 2.2.27+


Reference:
Stephen F. Altschul, Thomas L. Madden, Alejandro A. Schäffer,
Jinghui Zhang, Zheng Zhang, Webb Miller, and David J. Lipman (1997),
"Gapped BLAST and PSI-BLAST: a new generation of protein database
search programs", Nucleic Acids Res. 25:3389-3402.


Reference for
composition-based statistics:
Alejandro A. Schäffer, L. Aravind, Thomas L. Madden, Sergei
Shavirin, John L. Spouge, Yuri I. Wolf, Eugene V. Koonin, and
Stephen F. Altschul (2001), "Improving the accuracy of PSI-BLAST
protein database searches with composition-based statistics and
other refinements", Nucleic Acids Res. 29:2994-3005.


Database: nr
           71,551,133 sequences; 26,053,659,533 total letters


Query= G04373_P

Length=126
                                                                      Score     E
Sequences producing significant alignments:                          (Bits)  Value

emb|CDZ96876.1|  hypothetical protein [Xanthophyllomyces dendrorh...   239    3e-78
gb|EXI80474.1|  hypothetical protein AW10_01804 [Candidatus Accum...  37.7    1.5  
ref|WP_011288068.1|  hypothetical protein [Dechloromonas aromatic...  37.0    2.8  
ref|WP_052830431.1|  hypothetical protein [Gynuella sunshinyii] >...  35.0    3.5  
ref|WP_040085897.1|  protoheme IX farnesyltransferase [Corynebact...  36.2    5.8  
gb|AJE33245.1|  protoheme IX farnesyltransferase [Corynebacterium...  35.8    6.0  


 >emb|CDZ96876.1| hypothetical protein [Xanthophyllomyces dendrorhous]
Length=130

 Score =  239 bits (609),  Expect = 3e-78, Method: Compositional matrix adjust.
 Identities = 117/118 (99%), Positives = 118/118 (100%), Gaps = 0/118 (0%)

Query  8    EIKQQILGLDEKDEQERIRITALRDSRLAKRLQFVDIEEYHRRIFRKGDIENMDDGFGEI  67
            EIKQQILGLDEKDEQ+RIRITALRDSRLAKRLQFVDIEEYHRRIFRKGDIENMDDGFGEI
Sbjct  13   EIKQQILGLDEKDEQQRIRITALRDSRLAKRLQFVDIEEYHRRIFRKGDIENMDDGFGEI  72

Query  68   RAFHERRRREVTAFVWTSLLISVIGLSYNLWMISKYLIQTDWFRNLIGMKDPNAAISN  125
            RAFHERRRREVTAFVWTSLLISVIGLSYNLWMISKYLIQTDWFRNLIGMKDPNAAISN
Sbjct  73   RAFHERRRREVTAFVWTSLLISVIGLSYNLWMISKYLIQTDWFRNLIGMKDPNAAISN  130


>gb|EXI80474.1| hypothetical protein AW10_01804 [Candidatus Accumulibacter sp. 
BA-92]
Length=304

 Score = 37.7 bits (86),  Expect = 1.5, Method: Compositional matrix adjust.
 Identities = 31/100 (31%), Positives = 42/100 (42%), Gaps = 18/100 (18%)

Query  1    MPGKYHSEIKQQILGLDEKDEQERIRITA-------------LRDSRLAKRLQFVDIEEY  47
            +PGKYH       L  D++  QE  R T              L   R+ + L  V  E+ 
Sbjct  117  IPGKYHDFFSHHHLTFDQQSGQEEFRATVNRIFARNGVAFEMLSAGRIVRVLPPVLGEDL  176

Query  48   HRRIFRKGDIENMDDGFGEIRA-FHERR---RREVTAFVW  83
             R +FR GD   +D+   E RA F +R    RRE    +W
Sbjct  177  QRTVFRTGD-RTLDNMLDECRAKFSDRNPLVRREALERLW  215


>ref|WP_011288068.1| hypothetical protein [Dechloromonas aromatica]
 gb|AAZ47069.1| hypothetical protein Daro_2333 [Dechloromonas aromatica RCB]
Length=314

 Score = 37.0 bits (84),  Expect = 2.8, Method: Compositional matrix adjust.
 Identities = 32/100 (32%), Positives = 42/100 (42%), Gaps = 18/100 (18%)

Query  1    MPGKYHSEIKQQILGLDEKDEQERIRITA-------------LRDSRLAKRLQFVDIEEY  47
            +PGK+H       L  D++  QE  R T              L   R+ + L  V  EE 
Sbjct  117  IPGKHHDFFSHHHLTFDQQSGQEEFRATVNRIFSRNGVAFEMLSTGRIMRVLPPVLGEEL  176

Query  48   HRRIFRKGDIENMDDGFGEIRA-FHERR---RREVTAFVW  83
             R IFR GD   +D+   E RA F +R    RRE    +W
Sbjct  177  KRTIFRTGD-RTLDNMLDECRAKFSDRSPLVRREALERLW  215


>ref|WP_052830431.1| hypothetical protein [Gynuella sunshinyii]
 gb|AJQ96484.1| septum formation initiator [Gynuella sunshinyii YC6258]
Length=92

 Score = 35.0 bits (79),  Expect = 3.5, Method: Compositional matrix adjust.
 Identities = 15/47 (32%), Positives = 25/47 (53%), Gaps = 0/47 (0%)

Query  79   TAFVWTSLLISVIGLSYNLWMISKYLIQTDWFRNLIGMKDPNAAISN  125
            T F+W  L+++ IGL Y LW+  + L +    ++LI +K     I  
Sbjct  3    TKFLWAFLVVAFIGLQYRLWVGERSLAEAASLKSLIDLKKAQIEIQK  49


>ref|WP_040085897.1| protoheme IX farnesyltransferase [Corynebacterium humireducens]
Length=319

 Score = 36.2 bits (82),  Expect = 5.8, Method: Compositional matrix adjust.
 Identities = 21/59 (36%), Positives = 32/59 (54%), Gaps = 6/59 (10%)

Query  58   ENMDDGFGEIRAF----HERRRREVTAFVWTSLLISVI--GLSYNLWMISKYLIQTDWF  110
             ++D   G  RA     H    R+ T F W+ L+ISV+  GL  N W+ + ++I T+WF
Sbjct  72   SDIDQKMGRTRARPLVRHTVTNRKATIFAWSLLIISVLWLGLLANSWLAAFFIILTNWF  130


>gb|AJE33245.1| protoheme IX farnesyltransferase [Corynebacterium humireducens 
NBRC 106098 = DSM 45392]
Length=284

 Score = 35.8 bits (81),  Expect = 6.0, Method: Compositional matrix adjust.
 Identities = 17/42 (40%), Positives = 26/42 (62%), Gaps = 2/42 (5%)

Query  71   HERRRREVTAFVWTSLLISVI--GLSYNLWMISKYLIQTDWF  110
            H    R+ T F W+ L+ISV+  GL  N W+ + ++I T+WF
Sbjct  54   HTVTNRKATIFAWSLLIISVLWLGLLANSWLAAFFIILTNWF  95


Lambda      K        H        a         alpha
   0.329    0.143    0.434    0.792     4.96 

Gapped
Lambda      K        H        a         alpha    sigma
   0.267   0.0410    0.140     1.90     42.6     43.6 

Effective search space used: 640180337412


  Database: nr
    Posted date:  Sep 23, 2015 12:05 AM
  Number of letters in database: 26,053,659,533
  Number of sequences in database:  71,551,133


Matrix: BLOSUM62
Gap Penalties: Existence: 11, Extension: 1
Neighboring words threshold: 11
Window for multiple hits: 40
```
